# Characterization of the complete mitochondrial genome sequence of *Charybdis bimaculata* and phylogenetic analysis

**DOI:** 10.1080/23802359.2018.1524273

**Published:** 2018-10-05

**Authors:** Xue Liu, YiMing Yuan, YanLong He, ShouHai Liu, Xiao Ji, YuTao Qin, KeYi Ma

**Affiliations:** a National Demonstration Center for Experimental Fisheries Science Education, Shanghai Ocean University, Shanghai, People’s Republic of China;; b Key Laboratory of Exploration and Utilization of Aquatic Genetic Resources, Ministry of Education, Shanghai Ocean University, Shanghai, People’s Republic of China;; c Key Laboratory of Freshwater Aquatic Genetic Resources, Ministry of Agriculture, Shanghai Ocean University, Shanghai, People’s Republic of China;; d Shanghai Engineering Research Center of Aquaculture, Shanghai Ocean University, Shanghai, People’s Republic of China;; e East China Sea Environmental Monitoring Center of State Oceanic Administration, Shanghai, People’s Republic of China;; f Key Laboratory of Integrated Monitoring and Applied Technology for Marine Harmful Algal Blooms, SOA, Shanghai, People’s Republic of China

**Keywords:** *Charybdis bimaculata*, complete mitochondrial genome, phylogenetic analysis

## Abstract

In this study, the complete mitogenome of *Charybdis bimaculata* was sequenced and annotated. The mitogenome is 15,441 bp in length, containing 37 classical eukaryotic mitochondrial regions (13 typical protein-coding genes (PCGs), 22 tRNA genes, two rRNA genes) and a non-coding control region. Most of the genes are initiated with ATA, ATG, and ATT, though GTG is also used as an initiation codon. Twelve PCGs stop with complete termination codon TAA and TAG, while Cob uses incomplete codon (T–). The phylogenetic relationships based on 13 PCGs show that *C. bimaculata* clusters closest to *C. fariata* and *C. natator*.

In animals, the mitochondrial genomes (mitogenomes) are double-stranded circular, closed, and maternally inherited molecules (Boore 1999; Wolstenholme 1992). The mitogenomes can provide molecular data for further study on taxonomic status and conservation biology (Jiang et al. 2018). *Charybdis bimaculata* is a small portunid crab species recorded from Japan, Korea, China, Taiwan, Australia, India, and South Africa (Miyake 1983). It plays an important role in the benthic assemblage of the bay, serving as forage for demersal fishes (Kume et al. 1999; Yamaguchi and Taniuchi 2000). Therefore, we sequenced, annotated, and characterized its mitogenomes and used this to clarify its phylogenetic position.

The samples of *C. bimaculata* were collected from the East China Sea (120°33′27″E, 34°10′25″N). The specimen was stored in Sample Room of East China Sea Environmental Monitoring Center of State Oceanic Administration. Sequencing revealed that the full-length *C. bimaculata* mitogenome is a circular, double stranded molecule of 15,712 nucleotides in length (GenBank accession number MG787408). The *C. bimaculata* mitogenome contains the whole set of 37 genes (13 PCGs, 22 tRNA genes, and 2 rRNA genes), as well as a putative control region (non-coding region). The mitogenome of *C. bimaculata* exhibits a strong bias towards A and T nucleotides (33.95% A, 16.93% C, 37.64% T, and 11.48% G), with a total A + T content of 71.59%. The PCG region is 11,202 bp long, consisting of seven NADH dehydrogenase (NAD1–NAD6 and NAD4L), three cytochrome coxidase (cox1–cox3), two ATPase (ATP6 and ATP8) and one cytochrome b (Cob). Nine PCGs are coded on H-strand, while 4 PCGs are coded on L-strand. Most of the PCGs in *C. bimaculata* mitogenome start with a typical ATN (ATA, ATG, and ATT) codon, except ATP8 gene which starts with GTG. GTG is a specific initiation codon in the mitochondrial genome of eukaryotes (Zhang 1998). Twelve PCGs stop with complete termination codon (TAA and TAG), while Cob uses incomplete codon (T–) as termination codon.

On both sides of tRNA-Val, 16S rRNA (1310 bp) and 12S rRNA (833 bp) were located. All 22 tRNAs were identified in the *C. bimaculata* mitogenome and range from 64 to 76 bp in length. The non-coding region is located between the 12S rRNA and tRNA-Lle genes and has a length of 836 bp. Apart from the control region, eleven other small non-coding intergenic spacers were identified. Additionally, the *C. bimaculata* mitogenome has a total of 49 bp overlap between genes in 13 locations.

We established the phylogenetic relationships between 15 crabs based on the 13 PCGs amino acids using Neighbor-Joining methods (Figure 1). The results of the phylogenetic analysis revealed that *C. bimaculata* was closely clustered with other two previously reported species *Charybdis feriata* and *Charybdis natator* (Ma et al. 2015; Yang et al. 2017). In conclusion, the complete mitogenome of *C. bimaculata* provides essential and important molecular data for understanding the evolution and biogeography of *C. bimaculata*.

**Figure 1.**
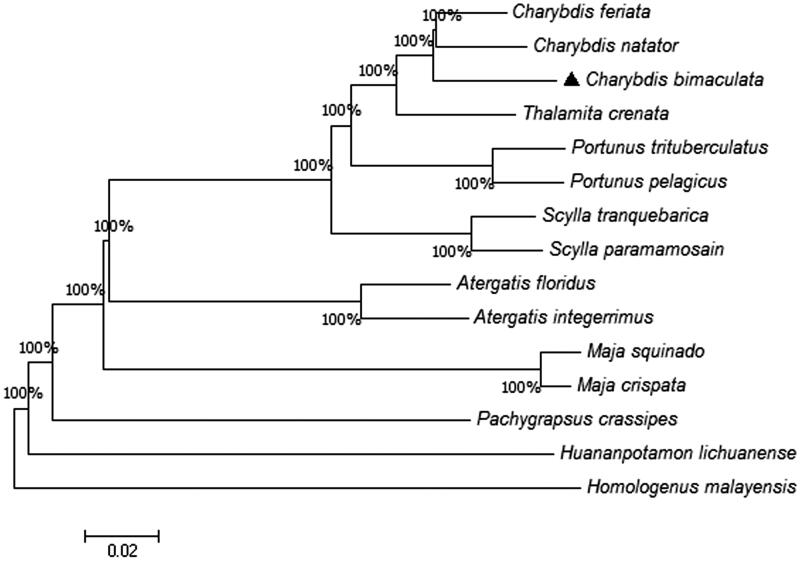
Phylogenetic tree inferred from the amino acid sequences of the 13 PCGs in the mitogenome. The complete mitochondrial genomes were downloaded from GenBank and the phylogenic tree was constructed by Neighbor-Joining method with 1000 bootstrap replicates.
